# Impact of Vitamin D Deficiency and VDR *TaqI* Polymorphism on Diabetic Retinopathy Risk Among T2DM Ethiopian Population

**DOI:** 10.1002/fsn3.70197

**Published:** 2025-04-21

**Authors:** Addisu Melake, Getachew Alamnie, Melaku Mekonnen

**Affiliations:** ^1^ Department of Biomedical Science, College of Health Science Debre Tabor University Debre Tabor Ethiopia; ^2^ Department of Biology, College of Natural and Computational Science Mekdela Amba University Tulu Awlya Ethiopia

**Keywords:** diabetic retinopathy, *TaqI*, type 2 diabetes mellitus, vitamin D deficiency

## Abstract

Many studies have shown that vitamin D deficiency and vitamin D receptor TaqI gene polymorphisms are associated with susceptibility to diabetic retinopathy in various populations. The objective of this study was to determine the impact of vitamin D deficiency and vitamin D receptor *TaqI* gene polymorphism on the risk of diabetic retinopathy complications in T2DM at the Debre Tabor Comprehensive Specialized Hospital, Northwest Ethiopia. 153 diabetic retinopathy patients and 153 healthy controls participated in an age‐ and sex‐matched hospital‐based case control study. To determine the related risk factors, demographic and clinical data were assessed. DNA was extracted from blood samples and subjected to polymerase chain reaction and agarose gel electrophoresis analysis to determine the *TaqI* genotypes. Vitamin D deficiency was detected in our investigation, and it was much more prevalent in patients than in controls (OR = 6.34, 95% CI = 3.85–10.42; *p <* 0.001). Moreover, both the *TaqI* tt genotype (OR: 2.18; 95% CI: 1.20–3.97; *p* = 0.010) and t allele (OR: 1.65; 95% CI: 1.19–2.30; *p* = 0.002) were substantially more prevalent in patients than in controls, indicating that it may be a major risk factor for the development of diabetic retinopathy. The findings point to a potential link between vitamin D deficiency and diabetic retinopathy complications. Moreover, *TaqI* gene polymorphisms have been linked to an increased risk of developing the disease in the Ethiopian population under study.

## Graphical abstract

Vitamin D deficiency was detected in our investigation, and it was much more prevalent in patients than controls (OR = 6.34, 95% CL = 3.85–10.42; *p <* 0.001). Moreover, both the *TaqI* tt genotype (OR: 2.18; 95% CL: 1.20–3.97; *p* = 0.010) and t allele (OR: 1.65; 95% CL: 1.19–2.30; *p* = 0.002) were substantially more prevalent in patients than in controls, indicating that it may be a major risk factor for the development of diabetic retinopathy.

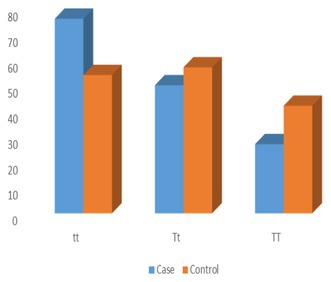

**Abbreviations**
BMI: Body Mass Index
CI: Confidence Interval
CL: Confidence Level
DBP: diastolic blood pressure
DNA: deoxyribonucleic acid
FBG: fasting blood glucose
HDL: high density lipoprotein
LDL: low density lipoprotein
OR: odds ratio
PCR: polymerase chain reaction
SBP: systolic blood pressure
T2DM: type 2 diabetes mellitus
*TaqI*
: ThermusaquaticusIThermus aquaticus I
TC: total cholesterol
TG: triglycerol
VDD: vitamin D deficiency
VDR: vitamin D receptor
WHR: Waist Hip Ratio

## 1 Introduction

Type 2 diabetes mellitus (T2DM) is a major public health concern affecting over 300 million people globally, resulting in severe morbidity and mortality (Luo et al. 2017). In addition to the disease's negative symptoms, long‐term complications can significantly reduce the quality of life of diabetes patients. Diabetic retinopathy (DR) is a common consequence of diabetes mellitus and the main cause of blindness in working‐age people (Nadri et al. 2019). Within 20 years of the disease's onset, over 60% of people with T2DM worldwide develop DR, with an estimated global prevalence of 34.6%; however, early detection can prevent blindness and severe vision loss (Senyigit 2019). According to recent estimates, the number of persons with diabetic retinopathy would reach 191 million by 2030. Many risk factors for DR have been established, such as hyperglycemia, high blood pressure, diabetes duration, diabetes starting age, hyperlipidemia, obesity, gender, and ethnicity (Luo et al. 2017). There is compelling evidence that adequate diabetes control helps to prevent DR, yet some people develop DR despite good control, implying that genetic factors influence vulnerability to retinopathy (Zhong et al. 2015).

Vitamin D, a secosteroid hormone, is obtained and synthesized through diet and exposure to UV radiation (Zhang et al. 2016). In addition to its well‐known role of maintaining calcium and phosphorus homeostasis, it exhibits powerful non‐classical features such as anti‐inflammatory, antioxidant, antiangiogenic, and antiproliferative effects. Vitamin D's non‐classical functions are gaining attention due to the close relationship between vitamin D deficiency and cancer, autoimmune diseases, type 2 diabetes mellitus (T2DM), metabolic syndrome, and diabetic retinopathy, as well as the prevalence of vitamin D deficiency (Lu et al. 2018). Vitamin D has been shown in studies to be a powerful inhibitor of retinal neovascularization and to reduce vascular endothelial growth factor (VEGF) production, indicating its role in the pathogenesis of diabetic retinopathy (Zhang et al. 2016). However, there is inadequate information to determine if blood vitamin D insufficiency is associated with diabetic retinopathy, and this association has been rarely investigated (Yuan et al. 2019).

The active form of vitamin D works on a specific vitamin D receptor (VDR), which is found in many human tissues and organs, including the retina. The VDR is a nuclear hormone receptor superfamily member that regulates target gene transcription and mediates vitamin D's genomic activities (Maia et al. 2016). The VDR gene is situated on human chromosomes 12q13‐12q14, and numerous single nucleotide polymorphisms (SNPs) have been identified, including *ApaI*, *BsmI*, *FokI*, and *TaqI* (Jiao et al. 2018). The *TaqI* polymorphism arises from a mutation in the transcriber region situated in exon 9 of the VDR gene (Liu et al. 2014). The mutation may impact VDR posttranscriptional regulation by interacting with microRNA; it is situated in the 3'‐untranslated region (UTR) of the VDR gene (Al‐darraji et al. 2017). Previous research on the relationship between VDR‐*TaqI* gene polymorphism and the risk of DR in Type 2 diabetes patients has been limited and inconsistent, with some indicating a novel association and others indicating no association, emphasizing the need for additional research (Jiao et al. 2018). Taking into account all of the previously mentioned variables, this study sought to determine the impact of vitamin D deficiency and *TaqI* gene polymorphism on the risk of diabetic retinopathy in the Ethiopian population.

## 2 Materials and Methods

### 2.1 Study Participants

A hospital‐based matched case–control study was carried out at Debre Tabor Comprehensive Specialized Hospital (DTCSH) from April to June 2024. At the chronic follow‐up clinic (CFC), the hospital treats and monitors patients with severe chronic illnesses such as DR and T2DM. The source population included all CFC patients, while the research participants were T2DM patients with DR problems who were being observed. The study's controls were age‐ and gender‐matched non‐diabetic, healthy volunteers from the same geographical location and social status who were available during the research period.

### 2.2 Inclusion and Exclusion Criteria

Type 2 diabetic patients with DR complications who had been confirmed by blood glucose tests and funduscopy were recruited into this study. The study included patients who had been receiving follow‐up care at CFC for at least 1 year. Controls were age‐ and sex‐matched non‐diabetic healthy individuals with normal blood glucose and funduscopy results from the same geographical location and social status. Patients with a history of chronic kidney disease, hyperparathyroidism, liver disease, vitamin D supplements, or chronic bacterial or viral infection were excluded. Patients who are unable to respond or are not willing to sign informed consent were also excluded from this study.

### 2.3 Sample Size Determination and Sampling Technique

The sample size was determined using the analytical study sample size calculation, with a confidence level of 95%, a power of 80%, and a double population proportion formula:

$$\text{Sample size}=\frac{r+1}{r}\frac{\left(p*\right)\left(1-p*\right)\;\left(Z_{\beta +Z1/2}\right)}{\left(P1-P2\right)2}$$

Because similar studies had not been conducted in the Ethiopian population, the sample size was calculated by assuming predicted proportions of 0.35 associations among the DR case group and 0.20 among the healthy control group. After accounting for the 10% non‐response rate, the final sample size was 306 participants of both sexes, including 153 DR patients and 153 healthy controls. Simple random sampling methods (TRN) were used to pick participants from all registered patients.

### 2.4 Anthropometric Measures

Body weight was assessed using a portable digital scale, and height was taken using a portable stadiometer. BMI was computed by dividing weight in kilograms by height in meters squared. Participants were classified as underweight (BMI < 18.5 kg/m^2^), healthy (18.5–25 kg/m^2^), overweight (25.0–29.9 kg/m^2^), or obese (≥ 30 kg/m^2^). The hip circumference was measured at the maximal circumference around the hips, and the waist circumference was measured at the umbilicus level with an inelastic measuring tape while the individuals stood upright. If the waist‐to‐hip ratio (WHR) was greater than 1.0, the participants were considered obese. Blood pressure was measured in the sitting position after 5 min of rest using digital equipment, and SBP and DBP were derived from the average of three measurements. The second and third measures were collected 5–10 min after the initial and second measurements, respectively. Participants were diagnosed with hypertension if their mean SBP was ≥ 140 mmHg and DBP was ≥ 90 mmHg, or if they used antihypertensive medication.

### 2.5 Biochemical Measures

Following an overnight fast, blood samples were collected from each participant's median cubital vein. After centrifuging the serum, each test was subjected to an enzymatic examination of glucose, triglycerides, total cholesterol, LDL‐cholesterol, and HDL‐cholesterol using the Dimension EXL 200 fully automated analyzer in the DTCSH diagnostic laboratory. Participants were classified as diabetes (FBG ≥ 126 mg/dL or RBG ≥ 200 mg/dL) or treated with insulin or oral hypoglycemic medications; pre‐diabetic (FBG 100–125 mg/dL or RBG 140–199 mg/dL); or normal (FBG < 100 mg/dL or RBG < 140 mg/dL). Dyslipidemia is defined as having triglycerides ≥ 150 mg/dL, total cholesterol ≥ 200 mg/dL, LDL‐C ≥ 130 mg/dL, and HDL‐C < 60 mg/dL. Serum vitamin D levels were evaluated using an enzyme‐linked immunosorbent assay (ELISA). The assay used was an automated platform assay (Immuno Diagnostic Systems Ltd., Bolden, Tyne and Wear, UK) and performed exactly as per the manufacturer's instructions. Briefly, the samples were subjected to a pretreatment step to denature the vitamin D‐binding protein. The treated samples were then neutralized in an assay buffer, and a specific anti‐25(OH)D antibody labeled with acridinium was added. Following an incubation step, magnetic particles linked to 25(OH)D were added. Following further incubation, the magnetic particles were “captured” using a magnet. After a washing step and the addition of trigger reagents, the light emitted by the acridinium label was inversely proportional to the concentration of 25(OH)D in the original sample. The concentration of 25(OH)D was calculated automatically using a 4‐point logistic curve. Vitamin D deficiency is defined as a 25‐hydroxyvitamin D (25‐OHD) level below 20 ng/mL (50 nmol/L).

### 2.6 Vitamin D Receptor Gene Analysis

The non‐enzymatic salting‐out method was employed to extract DNA from EDTA‐anticoagulated blood from both patients and controls. The *TaqI* genotypes were identified using forward primer 5'‐CAG AGC ATG GAC AGG GAG CAA‐3' and reverse primer 5'‐GCA ACT CCT CAT GGC TGA GGT CTC‐3'. We used the polymerase chain reaction (PCR) to determine the genotypes for the TaqI gene restriction fragment length polymorphism (RFLP). The initial denaturation stage of the PCR amplification was set to 95°C for 5 min, followed by 30 cycles of denaturation at 94°C for 60 s, annealing at 57°C for 45 s, extension at 72°C for 60 s, and final extension at 72°C for 7 min. The PCR‐RFLP involved digesting the PCR products using 1.5 μL of *Taq1* restriction enzyme at 65°C for 3 h. For electrophoresis, 5 μL of the digested reaction mixture was placed into a 2% agarose gel with ethidium bromide, run for 1 h at 120 V, and observed under UV illumination.

### 2.7 Statistical Analysis

The data was analyzed with STATA Version 17. To compare continuous variables between patients and controls, the t‐test for independent samples was applied. The genotype and allele frequency distributions were compared with the chi‐square test. Logistic regression was used to investigate the risk correlations of the *TaqI* polymorphism and vitamin D deficiency with DR at a 95% confidence level (CL). The *TaqI* genotypes, VDD, and clinical characteristics were evaluated using one‐way ANOVA. A *p*‐value of < 0.05 was considered statistically significant.

## 3 Results

### 3.1 Anthropometric and Biochemical Characteristics

The distribution of DR patient cases and non‐diabetic healthy control groups by gender and age was comparable. Of the 153 DR patients, 75 individuals (49.0%) were female, and 78 individuals (50.9%) were male. Similarly, among the 153 healthy control participants, 76 individuals (49.7%) were female, while 77 individuals (50.3%) were male. The case and control groups had mean ages of 59.6 ± 12.2 and 58.8 ± 10.3, respectively. Patients had significantly higher average levels of SBP, DBP, FBG, TG, TC, and LDL‐C compared to controls, but lower levels of HDL‐C and Vitamin D (*p* < 0.001). Body mass index (BMI) and waist‐hip ratio (WHR) were not significantly different between patients and controls (*p* > 0.05) (Table 1).

**TABLE 1:** Demographic and clinical characteristics.

Variables	Cases (*n* = 153)	Control (*n* = 153)	*p*
Sex (M/F)	78/75	77/76	0.7325
Age (years)	59.6 ± 12.2	58.8 ± 10.3	0.5378
BMI (kg/m^2^)	23.0 ± 4.3	22.8 ± 4.3	0.6634
WHR	0.78 ± 0.13	0.77 ± 0.11	0.3019
SBP (mmHg)	118.9 ± 6.4	114.1 ± 4.1	< 0.001*
DBP (mmHg)	77.4 ± 4.1	73.8 ± 4.1	< 0.001*
FBG (mg/dL)	141.7 ± 21.5	89.7 ± 8.7	< 0.001*
25(OH)D (ng/mL)	17.2 ± 7.0	24.4 ± 8.2	< 0.001*
Total Cholesterol (mg/dL)	181.6 ± 57.2	154.9 ± 49.0	< 0.001*
Triglyceride (mg/dL)	113.5 ± 70.9	111.5 ± 32.6	< 0.001*
LDL‐Cholesterol (mg/dL)	101.5 ± 36.2	85.1 ± 28.4	< 0.001*
HDL‐Cholesterol (mg/dL)	62.4 ± 10.6	68.5 ± 9.6	< 0.001*
VDD (%)	70.58	27.45	< 0.001*

* Statistically significant differences at *p* < 0.05.

### 3.2 Association Between Diabetic Retinopathy and Vitamin D Deficiency

The association between DR and VDD is shown in Table 2. A comparison of serum vitamin D levels between patients and controls found that patients with DR had lower levels of vitamin D than seemingly healthy control groups (*p* < 0.001). Patients also had a larger percentage of VDD (70.5%) compared to controls (27.4%).

**TABLE 2:** Association between diabetic retinopathy and vitamin D deficiency.

Vitamin D status	Cases (*n* = 153)	Control (*n* = 153)	OR (CI)	*p*
VDD	108 (70.6%)	42 (27.4%)	6. 34 (3.85–10.42)	< 0.001*
Non VDD	45 (29.4%)	111 (72.5%)	Ref	

* Statistically significant differences at *p* < 0.05.

### 3.3 Distribution of *TaqI* Genotypes and Allele Frequencies

**FIGURE 1:**
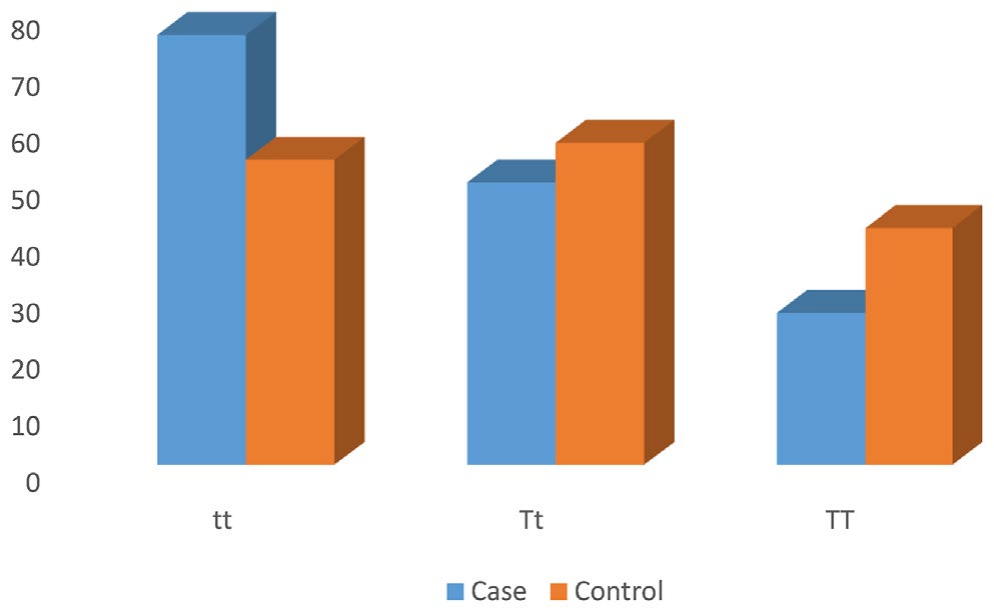
Distribution of the *TaqI* genotype in cases and controls.

The genotype distribution of *TaqI* gene polymorphisms is shown in Table 3. In DR patients, the frequencies of the tt, Tt, and TT genotypes were 49.7%, 32.7%, and 17.6%, respectively; in controls, the rates were 35.3%, 37.2%, and 27.4% (Figure 1). The homozygous tt genotype was found to be significantly more common in DR patients than in healthy controls (odds ratio [OR] = 2.18; 95% CI = 1.20–3.97; *p* = 0.010). The frequency of the t allele was substantially greater in cases compared to controls (OR = 1.65; 95% CI: 1.19–2.30; *p* = 0.002). The genotype distributions were not consistent with the Hardy–Weinberg equilibrium (*p* < 0.05) in both the patient (*χ* ^2^ = 11.29) and control (*χ* ^2^ = 9.58) groups. The t and T allele frequencies in patients were 0.66 and 0.34, respectively, while they were 0.54 and 0.46 in controls.

**TABLE 3:** Distribution of *TaqI* genotypes and allele frequencies.

	Cases (*n* = 153)	Control (*n* = 153)	OR (CI)	*p*
Genotype				
tt	76 (49.7%)	54 (35.3%)	2. 18 (1.20–3.97)	0.010*
Tt	50 (32.7%)	57 (37.2%)	1. 36 (0.73–2.52)	0.322
TT	27 (17.6%)	42 (27.4%)	Ref	
Allele Frequency				
t	202 (66.0%)	165 (53.9%)	1. 65 (1.19–2.52)	0.008*
T	104 (33.9%)	141 (46.1%)	Ref	

Abbreviations: CI, Confidence Interval; OR, Odds Ratio; Ref, Reference.

* *p*‐value < 0.05 is considered statistically significant.

### 3.4 Association Between *TaqI* Genotypes and Clinical Parameters

Table 4 summarizes the clinical parameters of DR patients and healthy controls according to the *TaqI* genotypes. The study groups TaqI genotypes (tt, Tt, and TT) were evaluated for BMI, WHR, blood pressure, fasting blood glucose, vitamin D levels, and lipid profiles. The tt genotype showed a stronger correlation with fasting blood glucose compared to the Tt and TT genotypes (123.0 ± 32.7 vs. 111.7 ± 28.4 and 106.8 ± 28.4; *p* = 0.0404). The genotypes in the research groups did not show a significant correlation with other clinical variables (*p* > 0.05).

**TABLE 4:** Association of *TaqI* genotype with clinical characteristics.

Variables	Genotypes			
	tt (*n* = 130)	Tt (*n* = 107)	TT (*n* = 69)	*p*
BMI (kg/m^2^)	22.8 ± 4.4	23.0 ± 4.5	22.7 ± 3.7	0.1645
WHR	0.78 ± 0.13	0.77 ± 0.12	0.77 ± 0.11	0.3068
SBP (mmHg)	116.7 ± 6.5	116.6 ± 5.3	115.9 ± 5.5	0.1198
DBP (mmHg)	75.7 ± 4.7	76.0 ± 4.1	74.6 ± 4.3	0.7523
FBG (mg/dL)	123.0 ± 32.7	111.7 ± 28.4	106.8 ± 28.4	0.0404*
25(OH)D (ng/mL)	20.5 ± 8.7	20.5 ± 8.6	21.6 ± 7.8	0.6254
Total Cholesterol (mg/dL)	167.5 ± 57.2	171.5 ± 53.3	164.4 ± 52.8	0.3026
Triglyceride (mg/dL)	127.1 ± 52.6	118.3 ± 41.9	120.1 ± 43.8	0.8829
LDL‐Cholesterol (mg/dL)	92.8 ± 32.0	98.7 ± 36.6	85.7 ± 29.9	0.4838
HDL‐Cholesterol (mg/dL)	66.0 ± 10.0	65.5 ± 11.3	64.2 ± 9.2	0.7381

* Statistically significant differences at *p* < 0.05.

## 4 Discussion

Emerging data from recent studies suggest that vitamin D may have an essential role in the development of diabetic retinopathy (DR), yet individual published investigations produced inconclusive results. Our study found that DR patients had significantly greater VDD compared to controls (OR = 6.34, 95% CI = 3.85–10.42; *p* < 0.001). These results were in line with studies from Korea (Jee et al. 2014), China (He et al. 2014), Spain (Alcubierre et al. 2015), United States (Millen et al. 2016), Turkey (Bener et al. 2018), India (Ashinne et al. 2018), and Iran (Afarid et al. 2020). The specific processes determining the influence of VDD on the occurrence of DR are still being investigated and can be due to various factors. Deficiency can limit nitric oxide generation, leading to inflammation and DR development. It can also exacerbate inflammation induced by hyperglycemia, resulting in retinal blood vessel leakage and abnormal angiogenesis (He et al. 2014). Oxidative stress can damage retinal blood vessels, diminish blood flow, and cause vision loss. Vitamin D deficiency may also affect insulin sensitivity and indirectly promote DR development by interacting with DR‐related biological processes. Vitamin D deficiency can impair blood pressure management and cause microvascular consequences like DR, with the importance of each pathway varying based on individual circumstances (Ashinne et al. 2018). Other risk factors include genetic vulnerability, diabetes duration, and glycemic management (He et al. 2014). Other studies undertaken in India (Reddy et al. 2015), Iran (Bonakdaran and Shoeibi 2015), England (Alam et al. 2016), and China (Zhao et al. 2021), however, found no significant relationship between VDD and the incidence of DR, contradicting the current study's conclusions. These contentious findings show the practical relevance of racial diversity and could be explained by a variety of factors, including sample size disparities, study methodology, selection bias, and matching criteria.

Studies on the relationship between vitamin D receptor (VDR) gene polymorphisms and diabetic retinopathy (DR) susceptibility yielded inconsistent results (Alhawari et al. 2022). In our study, individuals with DR showed much higher frequencies of the t allele of the *TaqI* variant than controls, as well as significantly higher rates of tt homozygosity (Table 3). The findings in French populations were consistent with the current study, as the VDR *TaqI* gene polymorphism showed a significant increase in the (tt) genotype and the (t) allele in DR patients compared to the control group (Taverna et al. 2020). The exact underlying mechanism by which the *TaqI* polymorphism in the VDR gene might cause DR is still not fully understood, and the evidence for a direct link is inconclusive (Alkhedaide et al. 2024). One proposed mechanism involves reduced vitamin D action, which may be due to less efficient VDR caused by the *TaqI* gene polymorphism. As a result, vitamin D may be less efficient in promoting insulin release by pancreatic beta cells and enhancing insulin sensitivity in target tissues (Alharbi et al. 2021). The impact of the *TaqI* gene polymorphism may also result from its probable interaction with other genetic and environmental variables, such as those who lead unhealthy lifestyles or who are genetically predisposed to DR (Alkhedaide et al. 2024). In contrast, a study of populations from Egypt (Ahmed et al. 2021), China (Song et al. 2019), and Jordan (Alhawari et al. 2022) found no link between the VDR *TaqI* polymorphism and the incidence of DR. The disparity between our findings and those of earlier studies could be attributed to genetic differences in the populations investigated or exposure to various environmental factors (Ahmed et al. 2021).

VDD is becoming better recognized as a substantial risk factor for T2DM‐related retinal outcomes. Nonetheless, it remains unclear if the development of VDD entails a complex interaction between genetic predisposition and clinical variables. As a result, we used serum vitamin D levels to evaluate the link between the *TaqI* gene polymorphism and VDD. Our findings indicated that there was no significant connection between VDD and the *TaqI* gene polymorphism (*p* > 0.05) (Table 4). There were still inconsistencies among researchers on the *TaqI* gene polymorphism in VDD, T2DM, and DR problems.

There are a few limitations to this study. First, the observed genotype distributions did not align with HWE, which is an important factor to consider when interpreting genetic association studies. The potential reasons for this deviation may be population stratification, small sample size, selection bias, or environmental influences specific to the Ethiopian population. Furthermore, while HWE deviations can impact the interpretation of genetic associations, our findings still provide valuable insights into the relationship between VDR TaqI polymorphism and diabetic retinopathy risk. We recommend cautious interpretation of the results and suggest further studies with larger sample sizes to validate our findings and explore the underlying reasons for the observed HWE deviation. Second, there were two groups in this study, DR patients and healthy controls. As a result, more study is needed, such as a third group, to definitively show the association between T2DM patients with no known history of DR, T2DM patients with DR problems, and non‐diabetic healthy controls. To the best of our knowledge, this is the first investigation into the relationship between genetic variations of *TaqI* and DR in the Ethiopian population. Although the results of this study will provide a baseline for these areas, more investigation is needed to identify additional gene polymorphisms that could be trustworthy markers of DR in this group.

## 5 Conclusion

Finally, our study found that vitamin D deficiency was substantially more common in DR patients than in controls, corroborating the previously documented association between low vitamin D and increased diabetic retinopathy risk in the Ethiopian population under study. Furthermore, there was a possible link between the *TaqI* gene polymorphism, namely the tt genotype and t allele, and diabetic retinopathy, implying that the *TaqI* gene polymorphism could be utilized as a biomarker to detect T2DM early, identify it, and prevent its complications. To completely understand the link between the VDD, *TaqI* gene, and DR, more research with a large sample size is needed.

## Author Contributions

The authors confirm contribution to the paper as follows: study conception and design: A.M. and G.A.; data collection: A.M., G.A., and M.M.; analysis and interpretation of results: A.M. and M.M.; draft manuscript: A.M. and G.A. All authors reviewed the results and approved the final version of the manuscript.

## Disclosure

The authors have nothing to report.

## Ethics Statement

The IRERC at the Debre Tabor University gave the study protocol approval (DTU/RE/305/2024) on March 18, 2024. Study participants were enrolled only after informed written consent was obtained from each participant. All of the information was collected in an anonymous manner and handled with care. The guiding principles of the Helsinki Declaration were followed throughout the entire data‐gathering process.

## Conflicts of Interest

The authors declare no conflicts of interest.

## Data Availability

The anonymised data used and/or analyzed in this study are available from the corresponding author upon reasonable request.
